# Construction and validation of a risk prediction model for oral frailty in elderly patients with chronic heart failure

**DOI:** 10.3389/fmed.2026.1775356

**Published:** 2026-04-13

**Authors:** Xue Song, Rong Chai, Jing Ye, Xiaohua Chen, Yuzhu Lin, Chen Xu

**Affiliations:** Mianyang Central Hospital, School of Medicine, University of Electronic Science and Technology of China, Mianyang, China

**Keywords:** chronic heart failure, elderly, nomogram, oral frailty, risk assessment model

## Abstract

**Objective:**

To investigate the prevalence of oral frailty among hospitalized elderly patients with chronic heart failure (CHF), identify its associated risk factors, and construct and validate a risk assessment model to provide scientific evidence for early identification and intervention.

**Methods:**

A convenience sample of 343 hospitalized elderly patients with chronic heart failure was recruited from a tertiary general hospital in Mianyang, China, between May and November 2025. Data were collected using a general information questionnaire, the Oral Frailty Index-8 (OFI-8), Frailty Phenotype (FP), Mini Nutritional Assessment–Short Form (MNA-SF), heart function–related clinical indicators, the Geriatric Oral Health Self-Efficacy Scale, the Geriatric Oral Health Assessment Index (GOHAI), and the Geriatric Depression Scale–Short Form (GDS-SF). Logistic regression analysis was performed to identify factors associated with oral frailty. A visualized nomogram prediction model was developed using R software. Model discrimination and calibration were evaluated using the receiver operating characteristic (ROC) curve, area under the curve (AUC), Hosmer–Lemeshow goodness-of-fit test, and Bootstrap resampling. Decision curve analysis (DCA) was conducted to assess the clinical applicability of the model.

**Results:**

A total of 350 questionnaires were distributed, and 343 valid questionnaires were returned, yielding an effective response rate of 98.0%. Among the 343 patients, 176 cases of oral frailty were identified, with a prevalence of 51.3%. Logistic regression analysis showed that advanced age, smoking, physical frailty, malnutrition, polypharmacy, and oral health–related self-efficacy were significant predictors of oral frailty (all *p* < 0.05). The prediction model demonstrated good discrimination, with an AUC of 0.857. The Hosmer–Lemeshow test indicated good model fit (*χ^2^* = 4.696, *p* = 0.790). After Bootstrap internal validation, the corrected concordance index (C-index) was 0.845, and the calibration curve showed good agreement between predicted and observed outcomes. Decision curve analysis indicated that the model provided a high net clinical benefit.

**Conclusion:**

The risk assessment model for oral frailty in elderly patients with chronic heart failure developed in this study demonstrates good discrimination and calibration. It may serve as a reliable tool for clinicians to identify and screen individuals at high risk of oral frailty at an early stage, thereby facilitating targeted prevention and intervention strategies.

## 1 Introduction

Chronic heart failure (CHF) is one of the most common chronic diseases among older adults and is characterized by high prevalence, high readmission rates, and high mortality. It is frequently accompanied by multiple health problems, including malnutrition, sarcopenia, and dysphagia (1, 2). Malnutrition is particularly common in patients with heart failure and has been shown to be associated with poor clinical outcomes, including increased hospitalization and mortality. In recent years, oral frailty (OF), defined as an age-related cumulative decline in oral structure and function, has attracted increasing attention worldwide. It is mainly manifested by tooth loss, poor oral hygiene, and impaired masticatory function (3, 4).

Accumulating evidence suggests that oral frailty is not only an important indicator of oral health in older adults but is also closely associated with malnutrition, sarcopenia, cognitive decline, and increased mortality. Moreover, oral frailty is considered an early and potentially reversible stage in the progression of physical frailty (5–7). Recent studies have also suggested that oral frailty may represent an important component of the broader frailty spectrum in older adults. Despite a reported prevalence ranging from 33.7% to 62.4% among older populations (8, 9), existing studies have predominantly focused on community-dwelling older adults or residents of long-term care facilities, with limited attention given to hospitalized elderly patients with CHF (10, 11).

Older patients with CHF are particularly vulnerable to oral frailty due to disease-specific characteristics and treatment regimens. Long-term use of diuretics, angiotensin-converting enzyme inhibitors (ACEIs), angiotensin receptor blockers (ARBs), angiotensin receptor–neprilysin inhibitors (ARNIs), and *β*-blockers may lead to xerostomia and reduced salivary secretion (12). In addition, impaired cardiac function, reduced physical capacity, and restricted nutritional intake may further exacerbate oral functional decline, increasing susceptibility to oral frailty in this population (13). Consequently, oral frailty may be more prevalent and detrimental among elderly patients with CHF, highlighting the importance of early identification of high-risk individuals to improve health outcomes and delay frailty progression.

Risk assessment models, which integrate multiple risk factors to quantify individual risk, have been widely applied in the cardiovascular field, including prediction of readmission and mortality risk. However, research on prediction tools specifically targeting oral frailty remains limited. Existing models are mainly developed for the general older population or community-dwelling individuals and fail to adequately capture the unique pathophysiological mechanisms and risk profiles of patients with heart failure, thereby limiting their clinical applicability. Therefore, developing a practical clinical tool to support early risk assessment and screening of oral frailty in elderly patients with CHF may help clinicians identify individuals who require further oral health evaluation and timely supportive interventions. Based on clinical data from hospitalized elderly patients with CHF, this study aimed to identify factors associated with oral frailty and to develop and internally validate a clinical model for risk assessment in this population, with the goal of facilitating early identification of vulnerable individuals and supporting targeted geriatric and nursing management.

## 2 Materials and methods

### 2.1 Study design and participants

This study was designed as a cross-sectional survey and conducted using a convenience sampling method. Elderly patients with chronic heart failure hospitalized at a tertiary general hospital in Mianyang, China, were recruited. The study protocol was approved by the Ethics Committee of the hospital (Approval No. S20250175-01). Written informed consent was obtained from all participants or their legal representatives prior to enrollment.

#### 2.1.1 Inclusion and exclusion criteria

A total of 350 elderly patients hospitalized between May and November 2025 were initially recruited.

The inclusion criteria were as follows:

1. Age ≥ 60 years;
2. Diagnosis of chronic heart failure according to the Chinese Guidelines for the Diagnosis and Treatment of Heart Failure (2024) (14);
3. Stable clinical condition with clear consciousness and ability to cooperate with assessments;
4. Voluntary participation with written informed consent.

The exclusion criteria were:

1. History of oral cancer or extensive oral/maxillofacial surgery resulting in significant anatomical alterations;
2. Long-term use of medications known to substantially affect frailty or oral health assessment;
3. Diagnosed Alzheimer’s disease, other forms of dementia, or severe psychiatric disorders;
4. Severe visual, hearing, or communication impairments preventing effective participation.

#### 2.1.2 Assessment of oral frailty

Oral frailty was assessed using the Oral Frailty Index-8 (OFI-8), developed by Tanaka et al. (15). The OFI-8 consists of eight items with a total score ranging from 0 to 11, with higher scores indicating poorer oral function. A cutoff value of 4 points yields both sensitivity and specificity of 80% (16).

### 2.2 Research methods

#### 2.2.1 Sample size estimation

Based on the events per variable (EPV) principle, an EPV of 10–20 was adopted to ensure model stability. Seventeen candidate predictor variables were included, and the expected prevalence of oral frailty was approximately 50%. Accordingly, the minimum required sample size was estimated to be 340 participants. Ultimately, 343 patients were included in the final analysis.

#### 2.2.2 Study variables and measurement instruments

Seventeen potential risk factors were identified through meta-analysis, expert consultation, and focused group discussions. The variables and measurement tools included:

1. General characteristics: age, sex, educational level, smoking status, alcohol consumption, and denture use. Age was categorized into clinically meaningful groups (60–69, 70–79, ≥80 years) to facilitate interpretation and application in bedside risk assessment. Continuous age was evaluated during preliminary analysis, and categorization did not significantly reduce predictive performance.
2. Disease and medication-related factors: New York Heart Association (NYHA) functional class, duration of heart failure, multimorbidity, polypharmacy, xerostomia, and chewing difficulty.
3. Physical frailty: assessed using the Frailty Phenotype (FP) proposed by Fried et al. (17), including unintentional weight loss, weak grip strength, slow walking speed, low physical activity, and fatigue. The presence of three or more criteria indicates frailty. The FP demonstrated a Cronbach’s *α* of 0.93 and a scale-level content validity index of 0.98 (18).
4. Nutritional status: evaluated using the Mini Nutritional Assessment–Short Form (MNA-SF), comprising six items with a total score of 14. Scores of 11–14 indicate normal nutritional status, while scores <11 indicate malnutrition. The sensitivity and specificity are 85.7% and 96%, respectively (19).
5. Depression: assessed using the 5-item Geriatric Depression Scale (GDS-5). Total scores range from 0 to 5, with higher scores indicating more severe depressive symptoms; a score ≥2 suggests the presence of depression. The scale has a sensitivity of 94% and specificity of 81% (20).
6. Oral health–related self-efficacy: measured using the Geriatric Self-Efficacy Scale for Oral Health (GSEOH), translated and validated by Xu Yuxin et al. (21). The scale includes 20 items across three dimensions: oral function, oral hygiene behaviors, and dental visit behaviors. Each item is rated on a 4-point Likert scale, with total scores ranging from 20 to 80; higher scores indicate higher oral health–related self-efficacy. The Cronbach’s *α* coefficient was 0.913.
7. Oral health status: assessed using the Geriatric Oral Health Assessment Index (GOHAI), translated by Wang Adan et al. (22). The GOHAI consists of 12 items covering three dimensions: physical function, psychosocial function, and pain or discomfort. Each item is rated on a 5-point Likert scale, yielding total scores from 12 to 60; higher scores indicate better oral health. The Cronbach’s *α* coefficient was 0.81.

#### 2.2.3 Data collection

Prior to the survey, a dedicated investigation team was established, and all members received standardized training and assessment. Trained investigators conducted face-to-face, one-on-one interviews using uniform instructions. For participants with low educational levels or visual impairment, investigators read the questionnaire items aloud and recorded the responses. All questionnaires were reviewed immediately after completion, and any missing items were supplemented on site to ensure data completeness and accuracy.

#### 2.2.4 Statistical analysis

Data analysis was performed using R software (version 4.4.1). Continuous variables were tested for normality. Normally distributed variables are presented as mean ± standard deviation (SD) and were compared using independent-samples t tests. Non-normally distributed variables are expressed as median (interquartile range, IQR) and were compared using the Mann–Whitney U test. Categorical variables are presented as frequencies and percentages, and comparisons between groups were conducted using the chi-square (*χ^2^*) test or Fisher’s exact test, as appropriate. A two-sided *p* value < 0.05 was considered statistically significant.

Variables that were statistically significant in univariable analyses were entered into a multivariable logistic regression model to identify factors independently associated with oral frailty. Prior to model construction, multicollinearity among candidate predictors was assessed using variance inflation factors (VIF). The linearity of continuous variables with the logit of the outcome was also examined. Based on the final regression model, a clinical model for assessing the risk of oral frailty was constructed and visualized as a nomogram using the RMS package in R. Model performance was evaluated in terms of discrimination and calibration. Discrimination was assessed using the receiver operating characteristic (ROC) curve and the area under the curve (AUC), while calibration was evaluated using the Hosmer–Lemeshow goodness-of-fit test. Decision curve analysis (DCA) was performed to evaluate the potential clinical utility of the model. Internal validation was conducted using bootstrap resampling with 1,000 repetitions to assess model stability and reduce the risk of overfitting.

## 3 Results

### 3.1 Baseline characteristics

The internal consistency of the OFI-8 in this study was moderate but acceptable, with a Cronbach’s *α* coefficient of 0.680. A total of 343 elderly patients with chronic heart failure were included, including 177 males and 166 females, with an age range of 60–86 years. Among them, 176 patients were identified as having oral frailty, yielding a prevalence of 51.3%. Detailed baseline characteristics of the participants are presented in Table 1.

**Table 1:** Baseline characteristics of hospitalized elderly patients with CHF.

Variable	Content	Number of cases (*n* = 343)
Age	60-69 years	127 (31.8%)
	70-79 years	137 (40.3%)
	≥80 years	79 (27.8%)
Gender	Male	177 (52.3%)
	Female	166 (47.7%)
Education level	Primary school or below	101 (33.5%)
	Junior high school	89 (29.0%)
	Senior high school/Secondary vocational school	103 (27.3%)
	College or above	50 (10.2%)
Smoking	No	217 (58.0%)
	Yes	126 (42.0%)
Alcohol consumption	No	220 (64.2%)
	Yes	123 (35.8%)
Number of chronic diseases	<3 types	134 (33.5%)
	≥3 types	209 (66.5%)
Polypharmacy	No	198 (52.3%)
	Yes	145 (47.7%)
Wearing dentures	No	160 (40.3%)
	Yes	183 (59.7%)
Xerostomia	No	167 (48.9%)
	Yes	176 (51.1%)
Malnutrition	No	240 (61.4%)
	Yes	103 (38.6%)
Chewing difficulty	No	256 (75.0%)
	Yes	87 (25.0%)
Depression	No	298 (84.1%)
	Yes	45 (15.9%)
Physical frailty	No	169 (41.5%)
	Yes	174 (58.5%)
Cardiac function classification	Grade I	94 (20.5%)
	Grade II	111 (31.8%)
	Grade III	84 (29.5%)
	Grade IV	54 (18.2%)
Duration of heart failure	<1 years	77 (22.2%)
	1-3 years	112 (31.3%)
	3-5 years	90 (26.7%)
	>5 years	64 (19.9%)

### 3.2 Univariable logistic regression analysis

The results showed that age, educational level, smoking status, multi morbidity, polypharmacy, malnutrition, NYHA functional class, physical frailty, GOHAI score, oral health–related self-efficacy, and denture use were significantly associated with oral frailty (all *p* < 0.05). Detailed results are presented in Table 2.

**Table 2:** Univariable analysis of oral frailty among hospitalized elderly patients with CHF.

Variable	Content	*n* (%)		Statistical metric	*p*
		Oral frailty group (*n* = 176)	Non-oral frailty group (*n* = 167)		
Age	60-69 years	56 (31.8%)	71 (42.5%)	6.292^a^	0.043
	70-79 years	71 (40.3%)	66 (39.5%)		
	≥80 years	49 (27.8%)	30 (18.0%)		
Gender	Male	92 (52.3%)	85 (50.9%)	0.065^a^	0.799
	Female	84 (47.7%)	82 (49.1%)		
Education level	Primary school or below	59 (33.5%)	42 (25.1%)	8.926^a^	0.030
	Junior high school	51 (29.0%)	38 (22.8%)		
	Senior high school/secondary vocational school	48 (27.3%)	55 (32.9%)		
	College or above	18 (10.2%)	32 (19.2%)		
Smoking	No	102 (58.0%)	115 (68.9%)	4.387^a^	0.036
	Yes	74 (42.0%)	52 (31.1%)		
Alcohol consumption	No	113 (64.2%)	107 (64.1%)	0.001^a^	0.980
	Yes	63 (35.8%)	60 (35.9%)		
Number of chronic diseases	<3 types	59 (33.5%)	75 (44.9%)	4.668^a^	0.031
	≥3 types	117 (66.5%)	92 (55.1%)		
Polypharmacy	No	92 (52.3%)	106 (63.5%)	4.405^a^	0.036
	Yes	84 (47.7%)	61 (36.5%)		
Wearing dentures	No	71 (40.3%)	89 (53.3%)	5.776^a^	0.016
	Yes	105 (59.7%)	78 (46.7%)		
Xerostomia	No	86 (48.9%)	81 (48.5%)	0.004^a^	0.947
	Yes	90 (51.1%)	86 (51.5%)		
Malnutrition	No	108 (61.4%)	132 (79.0%)	12.745^a^	<0.001
	Yes	68 (38.6%)	35 (21.0%)		
Oral health self-efficacy score		50.28 ± 6.020	57.51 ± 5.473	11.621^b^	<0.001
Oral health evaluation index score		45.41 ± 7.426	46.96 ± 4.366	2.339^b^	0.020
Chewing difficulty	No	132 (75.0%)	124 (74.3%)	0.025^a^	0.873
	Yes	44 (25.0%)	43 (25.7%)		
Depression	No	148 (84.1%)	150 (89.8%)	2.468^a^	0.116
	Yes	28 (15.9%)	17 (10.2%)		
Physical frailty	No	73 (41.5%)	96 (57.5%)	8.785^a^	0.003
	Yes	103 (58.5%)	71 (42.5%)		
Cardiac function classification	Grade I	36 (20.5%)	58 (34.7%)	11.543^a^	0.009
	Grade II	56 (31.8%)	55 (32.9%)		
	Grade III	52 (29.5%)	32 (19.2%)		
	Grade IV	32 (18.2%)	22 (13.2%)		
Duration of heart failure	<1 years	39 (22.2%)	38 (22.8%)	0.553^a^	0.907
	1-3 years	55 (31.3%)	57 (34.1%)		
	3-5 years	47 (26.7%)	43 (25.7%)		
	>5 years	35 (19.9%)	29 (17.4%)		

a: *X^2^* value, b: *t* value.

### 3.3 Construction of the oral frailty prediction model in elderly patients with CHF

Oral frailty (yes/no) was defined as the dependent variable. Variables that were statistically significant (*p* < 0.05) in univariable analysis were included as independent variables in a multivariable logistic regression model. Categorical variables were converted into dummy variables, and the assignment of independent variables is shown in Table 3.

**Table 3:** Assignment of variables in the multivariable logistic regression analysis.

Variable type	Variable name	Assigned value
Dependent	Oral Frailty	0 = No, 1 = Yes
Independent	Age	1 = 60-69 years, 2 = 70-79 years; 3 = ≥80 years
	Education level	1 = Primary school or below, 2 = Junior high school, 3 = High school/technical secondary school, 4 = College or above
	Smoking	0 = No, 1 = Yes
	Number of chronic diseases	0 = <3 types, 1 = ≥3 types
	Polypharmacy	0 = No, 1 = Yes
	Wearing dentures	0 = No, 1 = Yes
	Malnutrition	0 = No, 1 = Yes
	Physical frailty	0 = No, 1 = Yes
	Cardiac function classification	1 = Grade I, 2 = Grade II, 3 = Grade III, 4 = Grade IV

**Figure 1:**
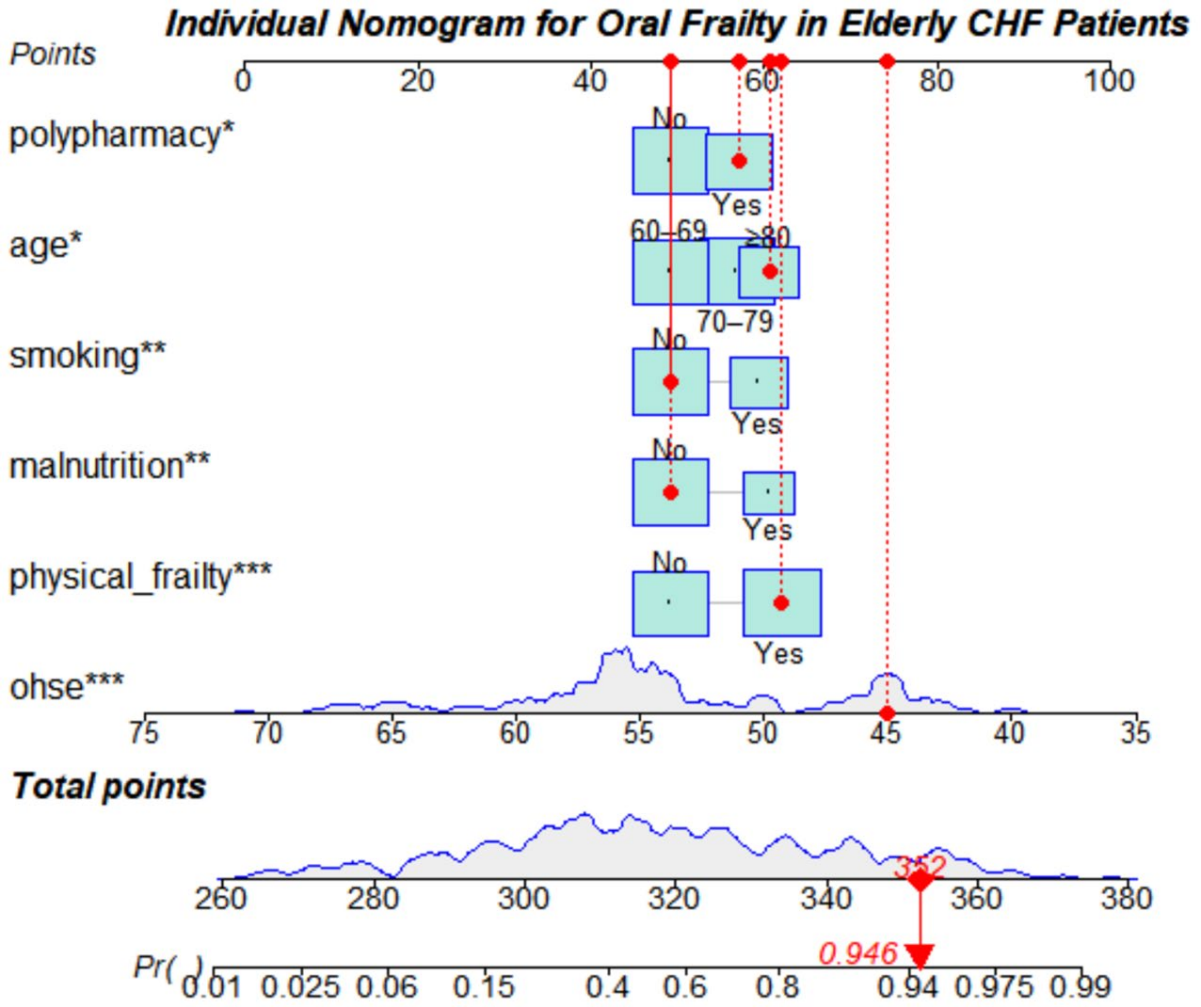
Nomogram for predicting oral frailty in elderly patients with CHF.

Prior to modeling, multicollinearity among candidate predictors was assessed using variance inflation factors (VIF), and all VIF values were below 5, indicating no significant multicollinearity. The linearity of continuous predictors with the logit of oral frailty was evaluated and confirmed to be appropriate. The results demonstrated that age, malnutrition, smoking status, polypharmacy, physical frailty, and oral health–related self-efficacy were independently associated with oral frailty in elderly patients with CHF (all *p* < 0.05) and were therefore incorporated into the final prediction model (Table 4). Based on the multivariate logistic regression results, the risk prediction model for oral frailty was expressed as follows: Logit (P) = 0.911 × Age + 0.814 × Smoking + 0.640 × Polypharmacy + 1.029 × Physical frailty − 0.911 × Malnutrition − 0.228 × Oral Health Self-Efficacy Score + 10.55 (intercept). A nomogram was subsequently developed using the rms package in R Studio, as illustrated in Figure 1.

**Table 4:** Multivariable logistic regression analysis of oral frailty in elderly patients with CHF.

Variable	B	SE	Wals	*p*	OR	95% CI	
						Lower	Upper
Age (≥80 years)	0.911	0.375	2.43	0.015	2.49	1.19	5.19
Smoking	0.814	0.290	2.81	0.005	2.26	1.28	3.98
Polypharmacy	0.640	0.285	2.24	0.025	1.90	1.08	3.32
Physical frailty	1.029	0.291	3.53	<0.001	2.80	1.58	4.95
Malnutrition	0.911	0.306	2.98	0.003	2.49	1.37	4.53
Oral health self-efficacy score	−0.228	0.028	−8.27	<0.001	0.80	0.75	0.84
Constant	10.55	1.464	7.21	<0.001			

B, Regression coefficient; SE, Standard error; Wals, Wals chi-square test statistic; OR, Odds ratio; CI, Confidence interval.

### 3.4 Performance evaluation of the prediction model

#### 3.4.1 Discrimination ability of the prediction model

**Figure 2:**
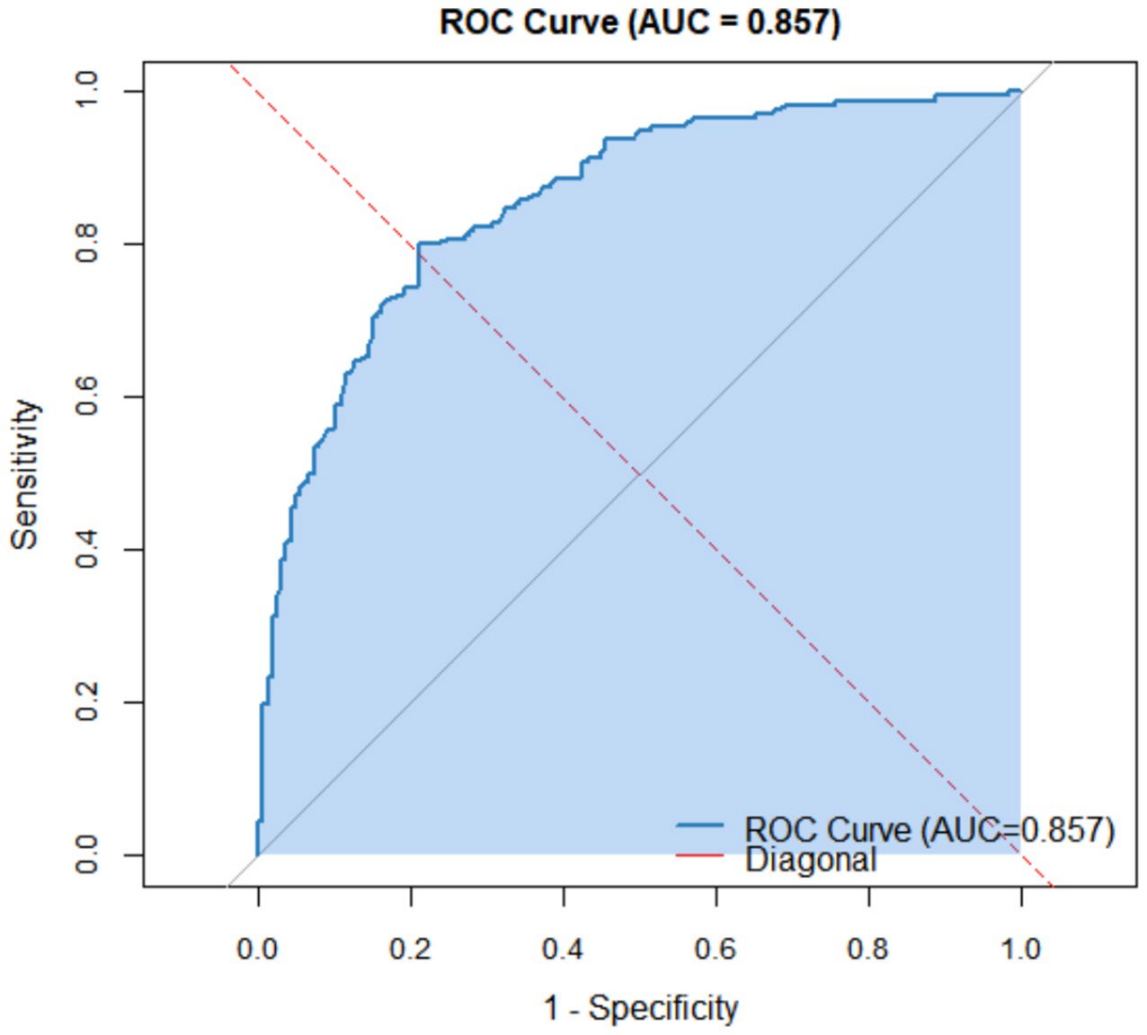
Receiver operating characteristic (ROC) curve of the risk assessment model.

Based on the multivariate logistic regression analysis, the receiver operating characteristic (ROC) curve of the oral frailty prediction model for elderly patients with chronic heart failure yielded an area under the curve (AUC) of 0.857 (95% CI: 0.818–0.896, *p* < 0.001), as shown in Figure 2. These results indicate that the model has good discriminatory ability and can effectively distinguish between patients with and without oral frailty.

#### 3.4.2 Calibration of the prediction model

The calibration of the prediction model was assessed using the Hosmer–Leme show goodness-of-fit test, which showed a *χ^2^* value of 4.696 with a *p* value of 0.790 (*p* > 0.05), indicating no significant difference between the predicted and observed outcomes. The calibration curve demonstrated good agreement between predicted probabilities and actual incidence, closely aligning with the ideal reference line, suggesting satisfactory model fit.

**Figure 3:**
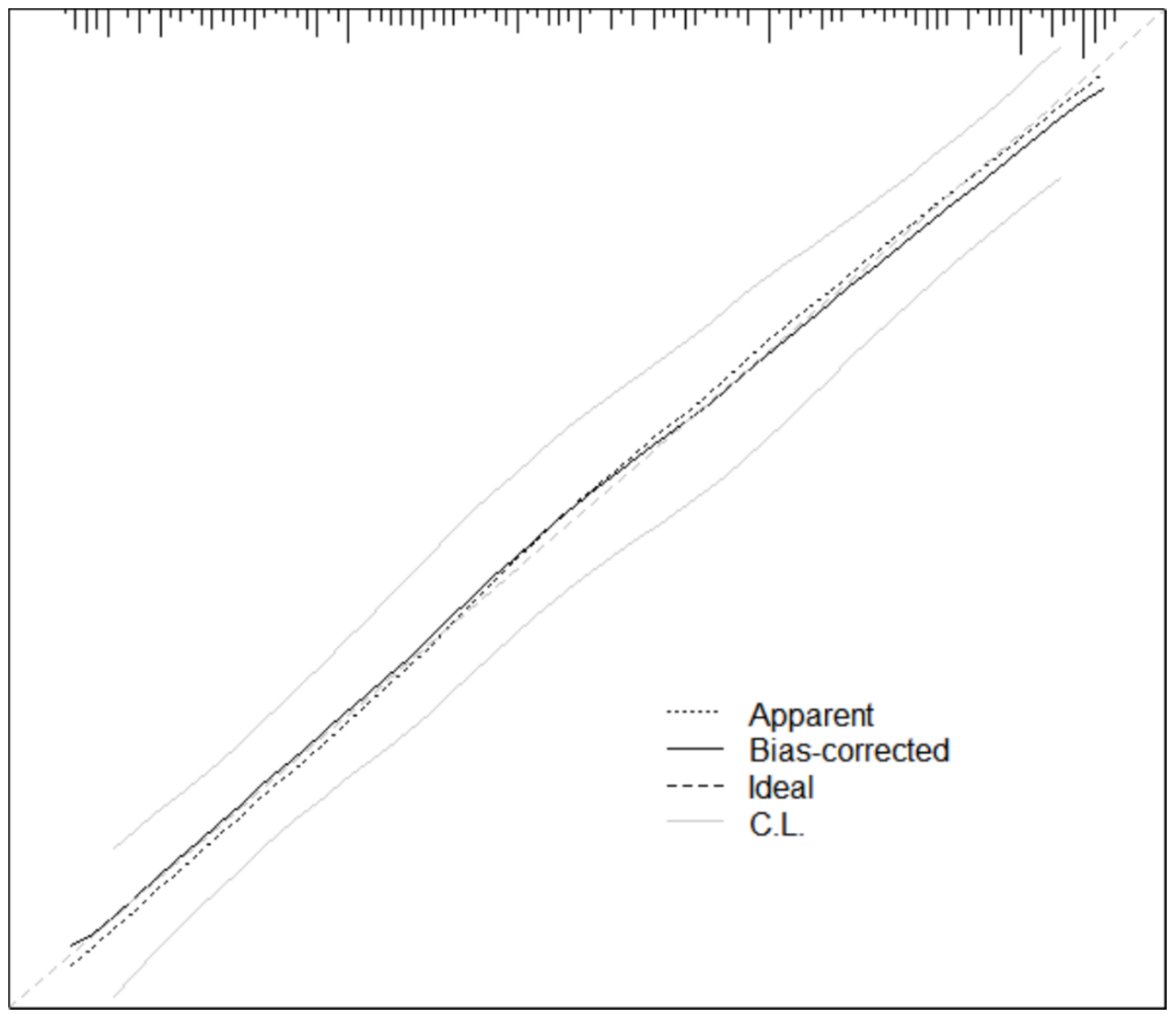
Calibration curve of the prediction model.

In addition, the Brier score was 0.153, further indicating a low overall prediction error and good calibration performance of the model. Detailed results are presented in Figure 3.

### 3.5 Internal validation of the prediction model

Internal validation was performed using the Bootstrap resampling method with 1,000 repetitions. The corrected concordance index (C-index) after validation was 0.845, which was comparable to the discrimination performance of the original model. These findings suggest that the model exhibits good stability, robustness, and generalizability in internal validation.

### 3.6 Clinical applicability of the prediction model

**Figure 4:**
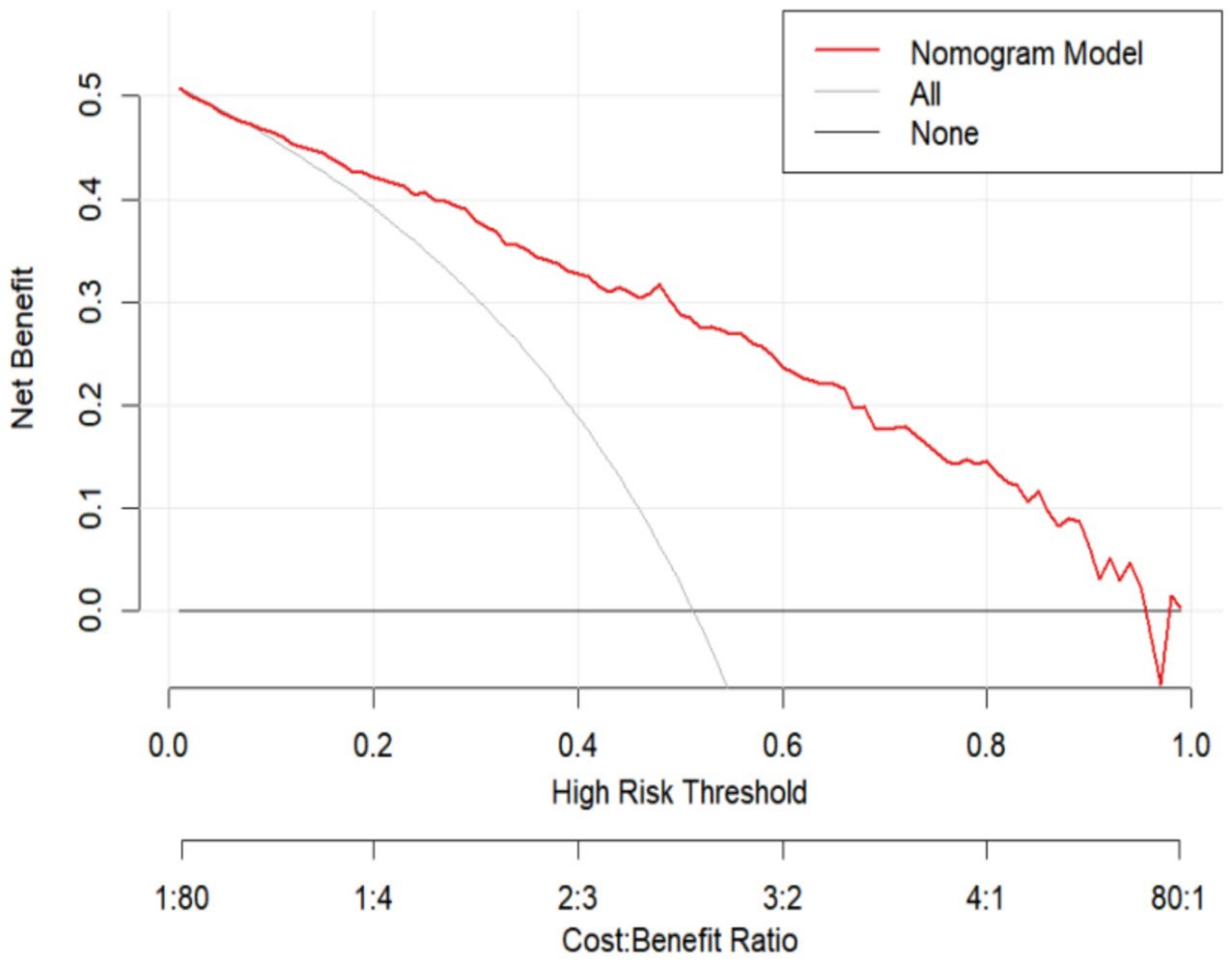
Decision curve analysis (DCA) of the prediction model.

Decision curve analysis (DCA) was performed to evaluate the clinical utility of the prediction model. The results demonstrated that the model provided a positive net benefit across a wide range of threshold probabilities (approximately 10%–95%), indicating good potential for clinical application (Figure 4). Patients classified as high risk according to the model may benefit from further oral health assessment, timely dental care, nutritional support, and targeted functional rehabilitation interventions. Therefore, the DCA results suggest that applying this model in clinical practice could support early identification of high-risk individuals and guide subsequent preventive or supportive management.

## 4 Discussion

### 4.1 Scientific rigor and clinical utility of the oral frailty risk assessment model in elderly patients with chronic heart failure

Based on clinical data from hospitalized elderly patients with chronic heart failure, this study constructed an oral frailty risk assessment model using multivariate logistic regression and visualized the model in the form of a nomogram. The model integrates demographic characteristics, clinical conditions, and oral health–related factors, which is consistent with the multifactorial and cumulative pathogenesis of oral frailty, thereby demonstrating strong scientific validity. Moreover, all variables included in the model are easily accessible in routine clinical practice, enhancing its feasibility and practicality for clinical application.

The performance evaluation indicated that the model has good discriminatory ability, with an AUC of 0.857, suggesting effective differentiation between patients with and without oral frailty. Calibration analysis demonstrated good agreement between predicted and observed probabilities, and the Bootstrap-corrected C-index of 0.845 further confirmed the model’s stability and generalizability. In addition, decision curve analysis revealed that the model yielded a positive net clinical benefit across a wide range of threshold probabilities (10%–95%), indicating favorable clinical utility for clinical risk identification and screening in elderly patients with CHF.

Compared with previous oral frailty prediction models that primarily targeted community-dwelling older adults or the general elderly population (23, 24), the present study specifically focused on elderly patients with chronic heart failure, a population at particularly high risk. By fully considering disease characteristics and treatment-related factors unique to CHF, the model is more closely aligned with real-world clinical scenarios. The nomogram format is intuitive and easy to use, enabling healthcare professionals to rapidly identify individuals who may require further oral health evaluation and supportive interventions during hospitalization.

These findings suggest that oral frailty in elderly patients with chronic heart failure is closely related to multiple geriatric conditions, including nutritional status, physical frailty, and oral health behaviors. The internal consistency of OFI-8 in this study was modest (*α* = 0.680), indicating that although the scale is useful for screening oral frailty, it may not fully capture all relevant dimensions. This may be related to the multidimensional nature of oral frailty and the clinical heterogeneity of hospitalized elderly patients with CHF. Therefore, the predictors identified in this study may reflect the broader frailty burden commonly observed in this population rather than disease-specific mechanisms of oral frailty. Consistent with previous studies, oral frailty may represent part of a broader geriatric vulnerability syndrome. Consistent with this perspective, the Heart Failure Association (HFA) frailty domains further highlight the multidimensional nature of frailty, emphasizing the importance of comprehensive assessment in patients with heart failure. Given the cross-sectional design, the model reflects associations at a single time point and is intended to support early risk identification and clinical screening rather than prediction of future clinical events.

### 4.2 Analysis of risk factors for oral frailty in elderly patients with CHF

This study identified advanced age as a significant risk factor for oral frailty in elderly patients with chronic heart failure. Consistent with the findings of Iwasaki et al. (25), the risk of oral frailty increases markedly among individuals aged 80 years and older. With advancing age, progressive deterioration of oral structures and functions occurs, including tooth loosening and loss, oral microbial imbalance, and worsening oral hygiene, all of which substantially increase the risk of oral frailty (26). Previous studies have shown that declining cognitive function and reduced activities of daily living impair older adults’ ability to maintain oral hygiene (27). Furthermore, age-related reductions in masticatory function may lead to salivary gland atrophy, gingival recession, and root exposure, thereby weakening oral self-cleansing and antimicrobial defenses. In addition, oral health literacy tends to decline with age, and older adults with lower health literacy are more susceptible to periodontal disease and tooth loss, accelerating the progression of oral frailty (28).

Physical frailty was also identified as a key factor associated with oral frailty. Existing evidence suggests that physical frailty and oral frailty are interrelated and may influence each other through malnutrition as a mediating factor (29, 30). In frail individuals, reductions in skeletal muscle mass and strength can affect not only limb muscles but also masticatory and swallowing-related muscles, resulting in impaired chewing efficiency and restricted food intake. Physical frailty is also closely linked to chronic inflammation, metabolic dysregulation, and reduced physical activity, which may collectively accelerate oral functional decline. As frailty progresses, problems such as chewing difficulty, dysphagia, and malnutrition become more pronounced, further compromising oral function and promoting the development of oral frailty (31, 32).

Malnutrition was another significant predictor of oral frailty in this study. Elderly patients with CHF are particularly prone to inadequate nutritional intake due to impaired cardiac function, reduced appetite, and dietary restrictions. Insufficient intake of essential nutrients required for maintaining oral mucosal integrity and dental structure may impair tissue repair capacity and increase susceptibility to oral frailty (33). Malnutrition not only compromises oral tissue metabolism and regeneration but also exacerbates muscle protein catabolism, further impairing chewing and swallowing function and creating a vicious cycle.

Polypharmacy was also identified as an important risk factor in the prediction model. Polypharmacy is common among elderly patients with chronic heart failure (34), who often require long-term combination therapy with diuretics, *β*-blockers, ACEIs/ARBs, or ARNIs. These medications may induce xerostomia, reduced salivary secretion, or taste disturbances, thereby disrupting the oral environment and diminishing oral self-cleaning capacity, which increases the risk of oral frailty (13). In addition, long-term polypharmacy may negatively affect medication adherence and indirectly influence oral care behaviors. Therefore, clinicians should consider the potential oral health consequences when managing polypharmacy, optimize medication regimens, and minimize unnecessary drug use whenever possible.

Smoking, as an unhealthy lifestyle factor, was also associated with oral frailty in this study. Smoking can alter salivary composition and oral microbiota, disrupt oral microecological balance, increase the risk of periodontal disease, and inhibit salivary secretion, thereby promoting deterioration of oral structure and function. Nicotine exposure reduces oral blood circulation, impairs tissue repair capacity, and facilitates the proliferation of pathogenic microorganisms, accelerating dental caries development (35, 36). In elderly patients with CHF, the cumulative effects of smoking may further amplify the risk of oral frailty. Therefore, healthcare professionals should strengthen health education, enhance patients’ awareness of the harmful effects of smoking, and promote healthy oral hygiene behaviors.

Oral health–related self-efficacy was found to be closely associated with oral frailty. Oral health–related self-efficacy reflects an individual’s confidence and ability to maintain oral health. Patients with lower self-efficacy are more likely to have inadequate oral hygiene practices, poor denture maintenance, and delayed dental visits, leading to the accumulation of oral problems and functional decline. In contrast, older adults with higher oral health–related self-efficacy tend to maintain better oral hygiene habits and engage in regular oral care, thereby reducing the risk of oral frailty (37).

Importantly, many of these factors may reflect the broader frailty burden commonly observed in elderly patients with chronic heart failure rather than disease-specific mechanisms alone. Furthermore, because oral frailty, malnutrition, and physical frailty may coexist and interact with each other, the associations observed in this cross-sectional study should be interpreted cautiously. Reverse causation and residual confounding cannot be completely excluded.

### 4.3 Study limitations

Several limitations should be acknowledged. First, this was a single-center study with a relatively limited sample size, which may introduce selection bias and restrict the generalizability of the findings. Second, some variables relied on clinical assessment or self-reported data, which may be subject to measurement bias. Third, the model was internally validated using bootstrap resampling, but external validation in independent populations is still required before broader clinical application. Future multicenter prospective studies incorporating larger samples, longitudinal follow-up, and more objective indicators are needed to further evaluate and refine the model.

## 5 Conclusion

This study developed a multidimensional risk assessment model for oral frailty in elderly patients with chronic heart failure. The nomogram provides a simple and practical tool for early identification of patients who may be at increased risk of oral frailty. By facilitating early screening and risk stratification, the model may help clinicians identify individuals who would benefit from further oral health evaluation and targeted supportive interventions.

## Data Availability

The original contributions presented in the study are included in the article/supplementary material, further inquiries can be directed to the corresponding authors.
